# Early versus delayed hip arthroplasty for femoral neck fractures in the elderly: a comparative study on multidimensional recovery

**DOI:** 10.3389/fmed.2026.1711563

**Published:** 2026-02-18

**Authors:** Feifan He, Feng Yang, Juan Wang, Yang Lu, Ganantes Ganati, Chong Gao, Jian Gao

**Affiliations:** 1 Department of Joint Surgery, The Sixth Affiliated Hospital of Xinjiang Medical University, Urumqi, China; 2 Department of Orthopedics, Nantong Rici Hospital Affiliated to Yangzhou University, Nantong, Jiangsu, China; 3 Department of Orthopedics, Affiliated Lianyungang Clinical College of Nantong University, Lianyungang, Jiangsu, China

**Keywords:** femoral neck fracture, forgotten joint score, GDS-15 scores, Harris score, hip arthroplasty, IADL scores, perioperative period, timing of surgery

## Abstract

**Background:**

The incidence of femoral neck fractures in the elderly is increasing due to global population aging, posing a significant public health challenge. The optimal timing for surgical intervention remains controversial. To determine if early surgical intervention reduces complications and enhances therapeutic efficacy in elderly patients with femoral neck fractures undergoing hip arthroplasty.

**Objective:**

To compare the effects of surgery performed ≤48 h (early) versus >48 h (late) after injury on 30-day complications and 1-year integrated somatic-psychosocial recovery.

**Methods:**

A retrospective cohort study enrolled 168 consecutive patients aged ≥65 years with Garden-IV femoral neck fracture who underwent hip arthroplasty between January 2023 and December 2024. 77 patients were operated on within 48 h and 91 after 48 h. The primary endpoint was the 30-day composite complication rate; secondary endpoints included length of stay (LOS), haemoglobin drop, inflammatory biomarkers, Harris Hip Score (HHS), Forgotten Joint Score (FJS), 15-item Geriatric Depression Scale (GDS-15) and Lawton Instrumental Activities of Daily Living (IADL) scale.

**Results:**

Early surgery reduced the 30-day composite complication rate to 29.9% versus 60.4% in the late group (χ^2^ = 15.670, *p* < 0.001, ARR = 30.5, 95%CI:(16.2 to 44.9%)), driven by lower incidences of hypoalbuminaemia (3.9% vs. 24.2%, χ^2^ = 13.542, *p* < 0.001, ARR = 20.3, 95%CI:(10.5 to 30.1%)) and joint pain (1.3% vs. 11.0%, χ^2^ = 6.401, *p* = 0.012, ARR = 9.7, 95%CI:(2.8 to 16.6%)). LOS was shortened by 4.6 days (t = −9.969, *p* < 0.001) and post-operative haemoglobin decline (115.43 ± 15.03 vs. 98.04 ± 18.48 g/L, *t* = 6.609, *p* < 0.001). At 1 month, the early group achieved 10.9 points higher HHS (79.12 ± 4.37 vs. 68.24 ± 8.06, *t* = 11.090, *p* < 0.001) and 13.3 points higher FJS (68.74 ± 7.10 vs. 55.46 ± 9.56, *t* = 10.308, *p* < 0.001); the advantage persisted at 3 months but disappeared at 6 months. GDS-15 scores were 2.2, 2.7 and 2.0 points lower at 1, 3 and 6 months (1 month: 5.40 ± 3.77 vs. 7.62 ± 2.49, *t* = −4.546, *p* < 0.001; 3 months: 2.99 ± 2.57 vs. 5.64 ± 1.74, *t* = −7.682, *p* < 0.001; 6 months: 1.95 ± 1.44 vs. 3.97 ± 2.21, *t* = −7.114, *p* < 0.001). Lawton-Brody IADL Scores (1 month: 26.29 ± 11.39 vs. 34.37 ± 3.75, *t* = −5.962, *p* < 0.001; 3 months: 23.27 ± 9.86 vs. 32.47 ± 4.17, *t* = −7.630, *p* < 0.001; 6 months: 20.84 ± 6.37 vs. 29.27 ± 8.06, *t* = −77.571, *p* < 0.001). No differences were observed in intra-operative blood loss, operative time, 90-day readmission or 1-year mortality.

**Conclusion:**

Hip arthroplasty performed within 48 h after femoral neck fracture in the elderly significantly decreases early complications, shortens hospitalisation, accelerates functional recovery and sustains better mood and daily activity without increasing intra-operative risk or late mortality.

## 1 Introduction

Hip fracture is a leading cause of morbidity and mortality in older adults, with femoral neck fractures accounting for a significant proportion of these injuries (1). As the global population ages, the incidence of femoral neck fractures is expected to rise substantially, posing a major challenge to healthcare systems worldwide (2). Current clinical guidelines recommend surgical treatment as the standard of care for displaced femoral neck fractures in elderly patients, with joint arthroplasty being the preferred modality due to its advantages in early mobilization and functional recovery (3, 4). Despite consensus on the need for surgical intervention, the optimal timing of surgery remains controversial. While some studies advocate for early surgery (within 24–48 h after injury) to reduce complications and improve outcomes (5–7), others argue that delaying surgery to allow for comprehensive preoperative optimization may be beneficial, especially in frail patients with multiple comorbidities (8–10). Previous research has primarily focused on short-term surgical outcomes, such as complication rates, length of hospital stay, and mortality. However, the broader impact of surgical timing on long-term functional recovery, psychological well-being, and social participation has received limited attention.

Elderly patients with femoral neck fractures often present with complex medical backgrounds, including cardiovascular disease, diabetes, and cognitive impairment (11, 12). Previous research has primarily focused on short-term surgical outcomes, such as complication rates, length of hospital stay, and mortality (13–15). However, the broader impact of surgical timing on long-term functional recovery, psychological well-being, and social participation—critical dimensions of comprehensive geriatric care—has received limited attention (16, 17). The evaluation of these multidimensional outcomes is particularly important, as they significantly influence treatment compliance and overall quality of life in this frail population.

Therefore, this study aimed to investigate the impact of early versus delayed hip arthroplasty on the comprehensive prognosis of elderly patients with femoral neck fractures. We hypothesized that early surgery (within 48 h) would be associated with lower complication rates, shorter hospitalization, improved functional outcomes, and better psychological and social recovery compared to delayed surgery.

## 2 Materials and methods

### 2.1 Inclusion criteria and exclusion criteria

Inclusion criteria: (1) Patients aged 65 years or older; (2) Those diagnosed with Garden type IV femoral neck fractures based on preoperative X-ray examination; (3) Those treated with hip arthroplasty; (4) Those willing to be included in the study and have signed the informed consent form.

Exclusion criteria: (1) Patients with open fractures; (2) Patients with missing preoperative X-ray and other imaging data; (3) Patients with hemiplegia; (4) Patients with a history of mental disorders in the past; (5) Patients with acetabular fractures; (6) Patients with pathological fractures caused by metastatic tumors or other reasons; (7) Patients with periprosthetic fractures; (8) Patients who refuse hip arthroplasty and choose other treatment methods.

### 2.2 General information

**Figure 1:**
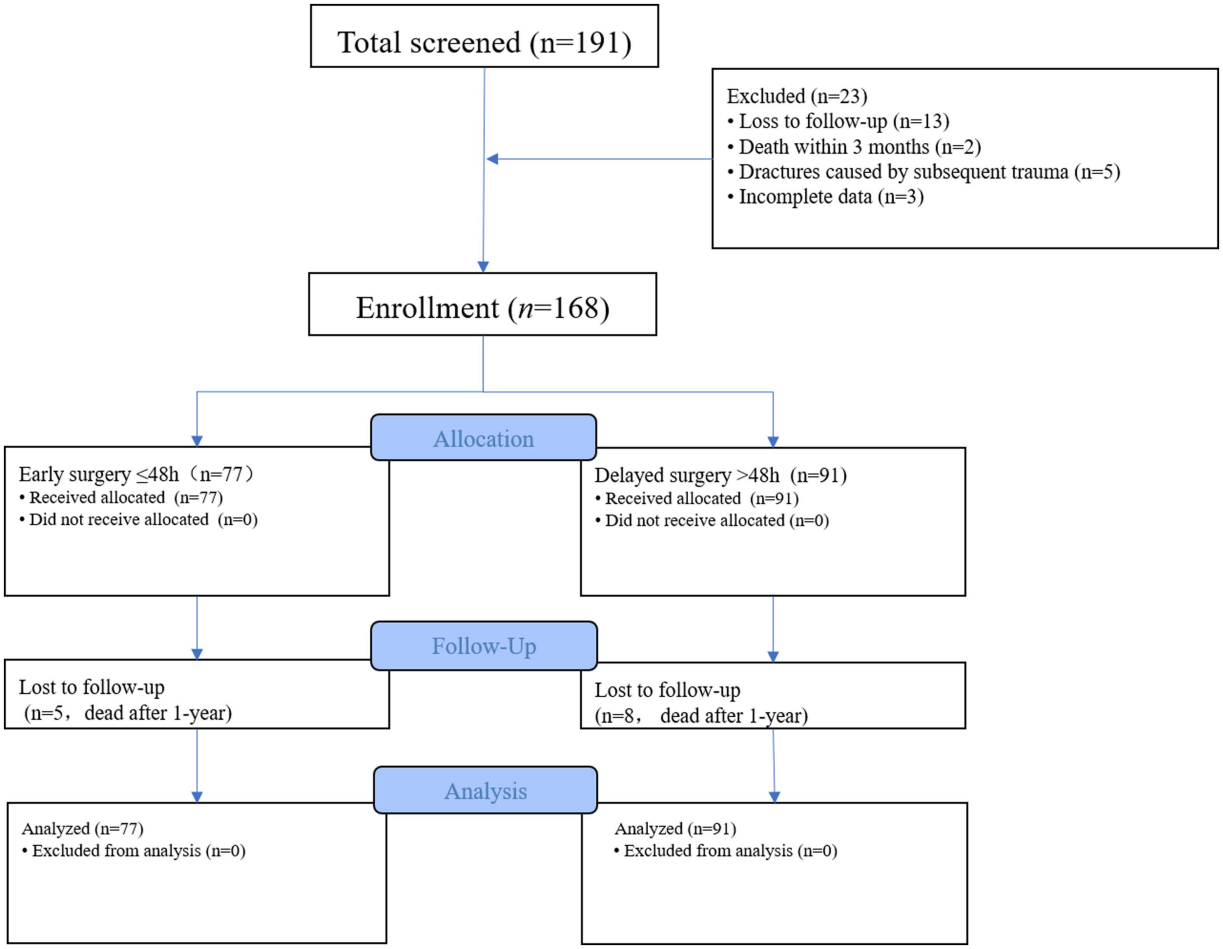
Flowchart of patient screening,and follow-up.

This study was a retrospective cohort study. Initially, 191 patients were included based on the inclusion criteria, while 23 cases were excluded (including 13 cases of loss to follow-up, 2 cases of death within 3 months, 5 cases of fractures caused by subsequent trauma, and 3 cases with incomplete data). Eventually, 168 patients were included (Figure 1).

The pre-injury comorbidities mainly included hypertension, diabetes, cardiovascular diseases, respiratory system diseases, with the incidence rates being 65.4% (104/168), 40.0% (62/168), 30.4% (51/168) and 41.7% (70/168), respectively. According to the different time intervals from injury to surgery, the patients were divided into two groups: the early group (EG) (77 cases after injury, 21 males and 56 females; age (77.05 ± 7.099) years); the late group (LG)(cases with injury time exceeding 48 h) 91 cases, 20 males and 71 females; age (78.91 ± 7.126) years; Primary Causes of Delay: Patient-related factors (*n* = 41): Many elderly patients sustained low-energy trauma with initially mild pain, leading to delayed recognition of fracture. These patients often self-managed with bed rest at home for 1–3 days before seeking medical attention. Inter-hospital transfer (*n* = 29): Patients underwent initial radiographs at local hospitals but were transferred to our tertiary center for surgical management due to concerns about operative risk and preference for specialized orthopedic care. Transfer processes and repeated consultations contributed to delay. Healthcare system factors (*n* = 21): This includes delays in diagnostic imaging appointments (especially during weekends/holidays), operating room scheduling conflicts, and preoperative clearance for patients with incomplete medical documentation upon arrival. There was no statistically significant difference in the general data between the two groups (*p* > 0.05), and the groups were comparable, as shown in Table 1.

**Table 1:** Comparative analysis of baseline characteristics between the two groups.

Characteristic	EG (*n* = 77)	LG (*n* = 91)	Statistical value	*p*-value
Age (years, Mean ± SD)	77.050 ± 7.099	78.910 ± 7.126	*Z* = −1.591	0.112
Gender (*n*)			χ^2^ = 0.634	0.426
Male	21	20		
Female	56	71		
Fracture Side (*n*)			χ^2^ = 0.338	0.642
Left	39	42		
Right	38	49		
ASA classification (*n*)			χ^2^ = 4.977	0.173
I, II	72	78		
III, IV	5	13		
Comorbidities (*n*)			χ^2^ = 2.724	0.099
Hypertension	43	61		
Diabetes	24	38		
Coronary heart disease	22	29		
COPD	29	71		
Surgical method (*n*)			χ^2^ = 3.652	0.070
Bipolar hemiarthroplasty	46	67		
Total hip arthroplasty	31	24		

### 2.3 Preoperative management

All patients were managed under a standardized, multidisciplinary care model. Upon admission, a multidisciplinary team comprising geriatricians, orthopedic surgeons, anesthesiologists, and rehabilitation specialists performed comprehensive geriatric assessments. These assessments included preoperative evaluation, cardiopulmonary and nutritional status, and fall risk. Geriatricians were directly involved in perioperative medical optimization, postoperative delirium prevention, and discharge planning. This approach ensured that the surgical process for elderly patients did not compromise the thoroughness of geriatric management.

### 2.4 Surgical method

All surgeries were performed by the same surgical team of physicians. According to the Anesthesia Practice Guidelines of the Canadian Society of Anesthesiologists (18), General anesthesia was prioritized for most patients; if the patient’s general condition was poor or if they were taking aspirin or clopidogrel long-term, general anesthesia or nerve block anesthesia was chosen. Surgical methods were selected based on the National Institute for Health and Care Excellence (NICE) guidelines and the consensus of experts in the diagnosis and treatment of elderly hip fractures, combined with imaging assessment, patient age, preferences, comorbidities, and surgical experience. Bipolar hemiarthroplasty (BH): 113cases; Total hip arthroplasty (THA): 55cases. During surgeries, products from Zhengtian Company were used: For bipolar hemiarthroplasty: bipolar femoral head prosthesis and biological femoral stem; for total hip arthroplasty: biological acetabular prosthesis, ceramic liner, biological stem, and ceramic femoral head.

### 2.5 Postoperative management

Postoperative general management: The affected limb should be externally rotated by approximately 15°, with a pillow placed between the legs. The drainage tube should be opened 6 h postoperatively and removed within 1–2 days.

Postoperative anticoagulation: According to the 2015 Chinese Guidelines for the Prevention of Venous Thromboembolism after Major Orthopedic Surgery, routine anticoagulation was administered to prevent lower limb thrombosis: 4250 IU of low molecular weight heparin sodium was injected subcutaneously once daily. After discharge, patients switched to oral rivaroxaban tablets 10 mg once daily (Bayer, USA).

Postoperative rehabilitation exercise: Patients were instructed to perform active contraction exercises of the ankle joint, gastrocnemius muscle, and quadriceps femoris on the bed, and underwent bilateral lower limb pneumatic compression therapy (30 min per session, twice daily) to prevent thrombosis.

Postoperative re-examination and evaluation of prosthesis position were conducted. After confirming good positioning, patients were instructed to perform bedside standing exercises, with hip joint movement not exceeding 90°. On the second day postoperatively, patients walked with the aid of a walker. After 3 months, when lower limb muscle strength recovered, patients could walk independently. Follow-up at 6 weeks, 1 month, 3 months, and 6 months postoperatively included imaging examinations, Harris Hip Score (HHS) (7) and Forgotten Joint score (FJS) (19).

### 2.6 Observational indices

#### 2.6.1 Clinical indices

(1) Baseline clinical data comparison: compare baseline clinical data, including demographic characteristics, comorbidities, and preoperative functional status, between the two groups.

(2) Perioperative outcomes:

Operative duration: total time from surgical incision to closure.
Intraoperative hemorrhage volume: quantified estimation of blood loss during surgery.
Blood transfusion volume: total volume of blood products administered intraoperatively and postoperatively.
Postoperative intensive care unit (ICU) admission rate: proportion of patients requiring critical care post-surgery.
Length of hospital stay: total days hospitalized from admission to discharge.
Hospitalization cost: total direct medical expenses incurred during hospitalization.
Inflammatory and infectious markers:
C-reactive protein (CRP): measured preoperatively and at multiple postoperative time points.
Complete blood count (CBC): including leukocyte count, hemoglobin, and platelet count.
Albumin: assess nutritional status and liver function.
Erythrocyte sedimentation rate (ESR): assessed to evaluate systemic inflammation.
Procalcitonin (PCT): quantified to monitor sepsis or bacterial infection.

Postoperative complications:

Infection: Including surgical site infections (SSIs) and systemic infections. The severity of all infectious complications was classified using the Clavien-Dindo grading system.
Dislocation: Incidence of prosthetic hip joint dislocation. Dislocation was defined as the complete separation of the femoral head component from the acetabular socket, occurring within 30 days after surgery, and requiring clinical intervention.
Hip joint pain: Hip joint pain was defined as new-onset or significantly worsened pain in the operated hip, assessed using the Visual Analog Scale (VAS). A clinically meaningful complication was predefined as a reported VAS score ≥4 out of 10 at any assessment point within the 30-day postoperative period.
Prosthesis loosening: Prosthesis loosening was defined as radiographic evidence of implant instability within the 30-day postoperative period, based on standardized anteroposterior and lateral hip radiographs.
Heterotopic ossification: Heterotopic ossification (HO) was defined as the radiographic evidence of new bone formation in the peri-articular soft tissues, assessed on standard anteroposterior pelvic radiographs taken within the 30-day postoperative period. Graded using the Brooker classification system.
Hypoproteinemia: Hypoproteinemia was defined as a serum total protein level below 60 g/L (6.0 g/dL) at any point within the 30-day postoperative period, based on standard laboratory reference ranges.
Deep vein thrombosis (DVT): Diagnosed via Doppler ultrasound or venography.

(3) Postoperative functional outcomes:

Follow-up assessments: Conducted at 6 weeks, 1 month, 3 months, 6 months and 1 year postoperatively.
Hip joint function: Evaluated using the Harris Hip Score (HHS) (7) and Forgotten Joint score (FJS) (19) to assess functional recovery and patient-reported outcomes.
Psychological status (15-item Geriatric Depression Scale [GDS-15]), and social participation (Lawton IADL) at identical intervals.

#### 2.6.2 Radiological indices

All patients underwent postoperative radiographic evaluation within 24 h post-surgery. Key radiological parameters included:

Neck-shaft angle: Angular measurement between the femoral neck and femoral shaft.
Lateral eccentricity: Horizontal offset of the prosthetic femoral head relative to the acetabular component.
Vertical center of rotation: Vertical height of the prosthetic joint’s rotational center relative to anatomical landmarks.
Engh score: Radiographic assessment of prosthetic integration and osseointegration (20, 21).

### 2.7 Statistical analysis

Data analysis was conducted using SPSS 26.0. Measurement data were expressed as x¯ ± s. For normally distributed data, independent sample t-test was used with a two-tailed test. For non-normally distributed data, Mann–Whitney U test was used. For count data or categorical variables, χ^2^ test or Fisher’s exact test was employed. *p* < 0.05 was considered statistically significant.

## 3 Results

### 3.1 Baseline characteristics

A total of 168 elderly patients with femoral neck fractures were included in this retrospective cohort study. Among them, 77 patients underwent early surgery within 48 h post-injury (early group), and 91 patients received delayed surgery more than 48 h after injury (delayed group). No significant differences were observed between the two groups in terms of age, sex, fracture side, ASA classification, comorbidities, surgical approach, laboratory parameters (all *p* > 0.05), indicating good comparability (Tables 1, 2).

**Table 2:** Comparative analysis of laboratory parameters between the two groups.

Laboratory parameters	EG (Mean ± SD)	LG (Mean ± SD)	t-value	*p*-value
pH value	7.401 ± 0.031	7.406 ± 0.388	−0.989	0.324
pCO₂ (mmHg)	38.819 ± 3.660	37.479 ± 3.823	2.303	0.063
pO₂ (mmHg)	75.155 ± 20.045	75.513 ± 20.951	−0.113	0.911
Hematocrit (%)	44.1026 ± 40.338	44.851 ± 9.522	−0.487	0.627
Oxygen saturation (%)	93.355 ± 4.153	93.397 ± 3.812	−0.067	0.947
Residual alkalinity (mmol/L)	−0.772 ± 1.825	−1.048 ± 1.998	0.918	0.36
Bicarbonate (mmol/L)	23.710 ± 1.631	23.401 ± 1.797	1.160	0.248
Lactic acid (mmol/L)	2.157 ± 0.858	2.111 ± 0.743	0.362	0.718
Leukocyte count (10^9^/L)	7.683 ± 2.232	7.795 ± 2.398	−0.313	0.755
Red blood cell count (10^12^/L)	4.183 ± 0.511	4.116 ± 0.517	0.844	0.400
Hemoglobin (g/L)	126.380 ± 16.259	125.080 ± 16.455	0.514	0.608
Albumin (g/L)	42.710 ± 6.585	41.230 ± 8.623	1.235	0.219
Platelet count (10^9^/L)	19567.890 ± 6282.319	21316.820 ± 5700.006	−1.869	0.063
Neutrophil count (10^9^/L)	5.748 ± 2.129	5.915 ± 2.344	−0.480	0.632
Lymphocyte count (10^9^/L)	1.234 ± 0.436	1.173 ± 0.451	0.891	0.374
Monocyte count (10^9^/L)	0.573 ± 0.221	0.554 ± 0.224	0.553	0.581
Eosinophil count (10^9^/L)	0.103 ± 0.787	0.130 ± 0.108	−1.787	0.076
Basophil count (10^9^/L)	0.027 ± 0.014	0.026 ± 0.017	0.314	0.754
Prothrombin time (s)	11.969 ± 0.6916	11.983 ± 0.800	−0.122	0.903
Prothrombin time activity (%)	81.448 ± 0.648	83.335 ± 13.841	−1.012	0.313
INR	1.041 ± 0.061	1.042 ± 0.071	−0.113	0.910
APTT (s)	28.876 ± 2.825	29.426 ± 3.002	−1.228	0.221
Fibrinogen (g/L)	3.703 ± 0.950	3.848 ± 1.057	−0.938	0.350
Thrombin time (s)	17.071 ± 1.768	17.244 ± 1.682	−0.658	0.511
D-Dimer (mg/L)	4.201 ± 4.353	4.411 ± 5.638	−0.271	0.797
Antithrombin III activity (%)	85.285 ± 10.635	82.631 ± 12.361	1.501	0.135

### 3.2 Intraoperative outcomes

**Figure 2:**
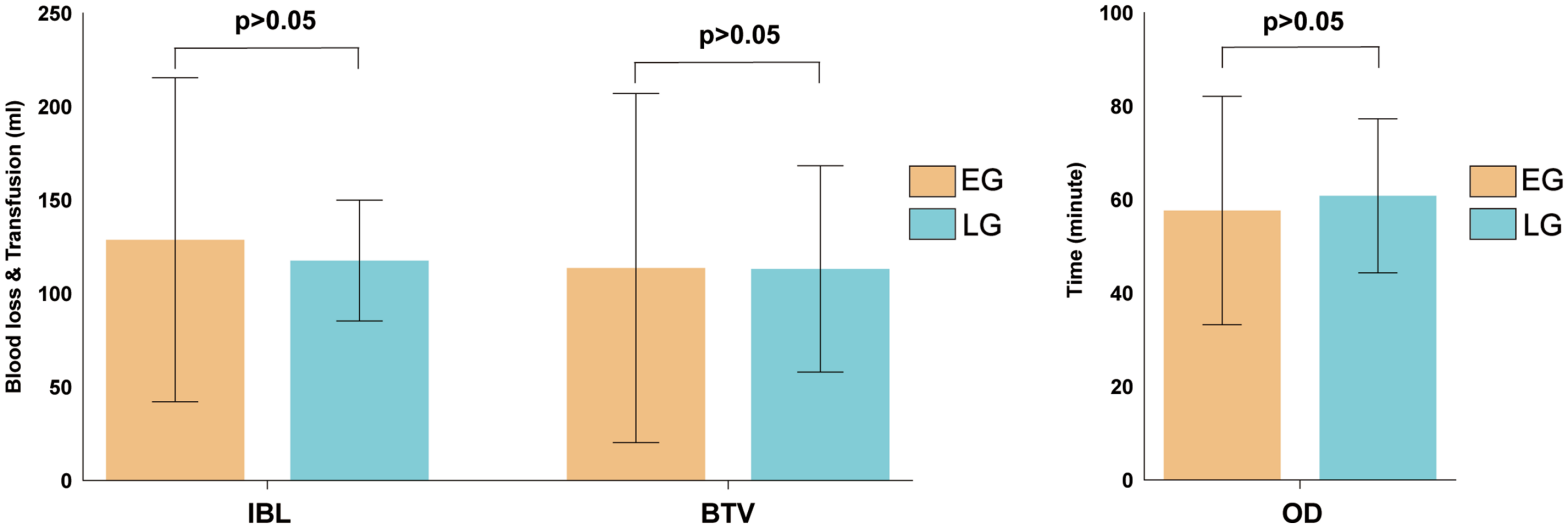
Comparative analysis of intraoperative outcomes between the two groups.

There were no statistically significant differences between the early and delayed groups in terms of intraoperative blood loss (120.32 ± 91.38 mL vs. 117.64 ± 32.32 mL, *t* = −0.965, *p* = 0.336), intraoperative transfusion volume (120.78 ± 122.03 mL vs. 113.19 ± 55.19 mL, *t* = 0.245, *p* = 0.807), or operative time (57.66 ± 24.44 min vs. 60.82 ± 16.49 min, *t* = 0.504, *p* = 0.615) (Figure 2).

### 3.3 Radiological assessment

Postoperative radiographic evaluation within 24 h revealed no significant differences between the two groups in terms of neck-shaft angle (NSG), horizontal Offset (HO), vertical center of rotation (VCR), leg length (LL)(*p* > 0.05). Additionally, Engh scores (ES), used to assess femoral stem fixation and stability, showed comparable results between the groups (*p* > 0.05). Detailed findings are presented in Table 3.

**Table 3:** Radiological assessment of patients between the two groups.

Parameter	EG (Mean ± SD)	LG (Mean ± SD)	t-value	*p*-value
NSG (°)	130.694 ± 2.356	130.338 ± 2.341	−1.040	0.300
HO (mm)	44.087 ± 3.185	43.528 ± 3.673	−1.123	0.263
VCR (mm)	63.184 ± 2.786	63.177 ± 2.559	−0.016	0.987
LL (mm)	3.370 ± 3.308	2.751 ± 3.314	−1.328	0.186
ES (Fixation)	9.692 ± 1.584	9.723 ± 1.598	0.001	0.999
ES (Stability)	15.201 ± 0.980	15.342 ± 1.048	0.936	0.351
Overall ES	24.920 ± 1.744	25.063 ± 1.894	0.522	0.602

### 3.4 Inflammatory markers

**Figure 3:**
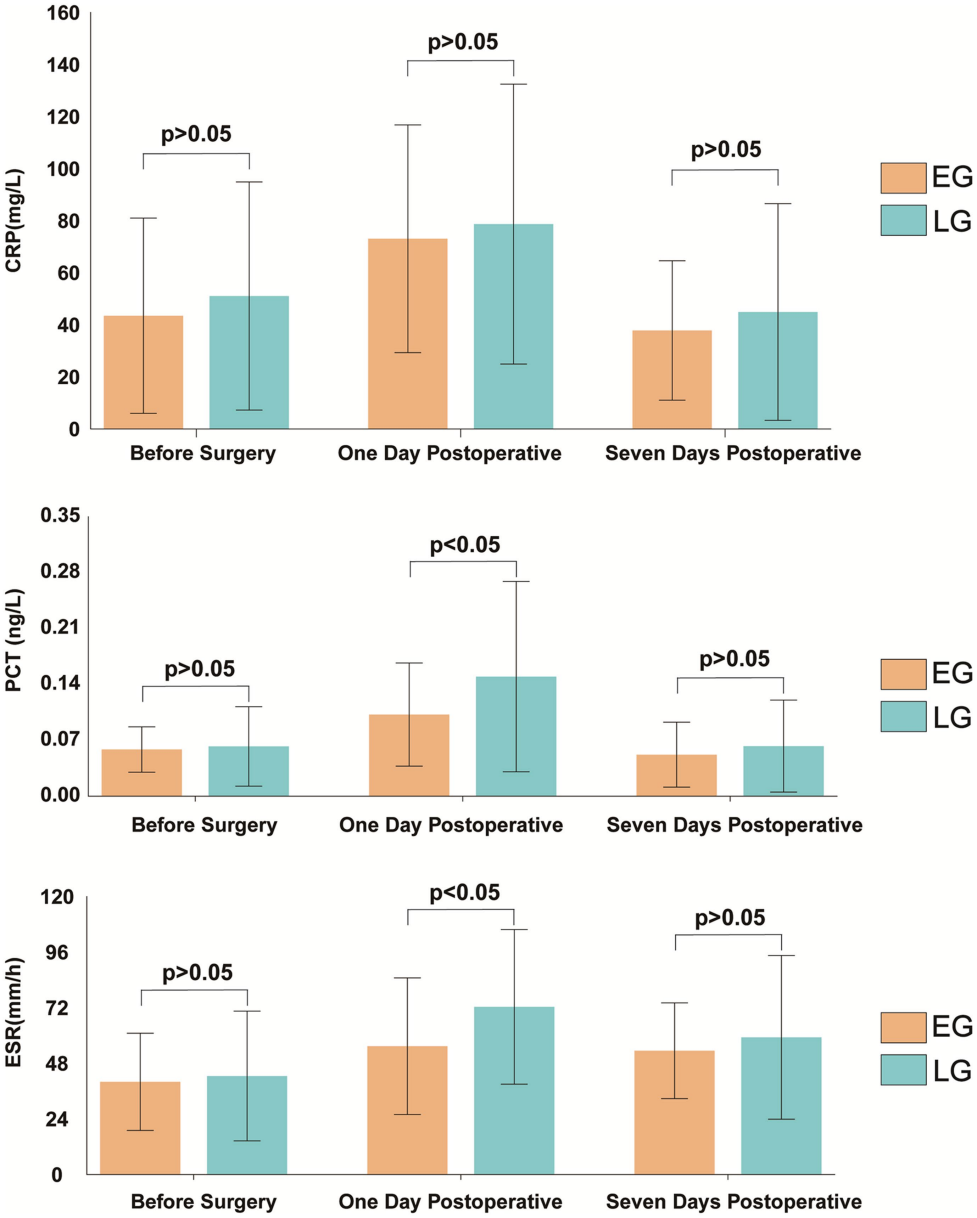
Inflammatory marker levels between the two groups.

Preoperative levels of C-reactive protein (CRP), procalcitonin (PCT), and erythrocyte sedimentation rate (ESR) were similar between groups. On postoperative day 1, PCT and ESR were significantly lower in the early group (PCT: 0.102 ± 0.065 vs. 0.150 ± 0.119 ng/mL, *t* = −3.272, *p* = 0.001; ESR: 55.39 ± 29.48 vs. 72.35 ± 33.38 mm/h, *t* = −3.461, *p* < 0.001). By postoperative day 7, these differences were no longer statistically significant (all *p* > 0.05) (Figure 3).

### 3.5 Hemoglobin levels

**Figure 4:**
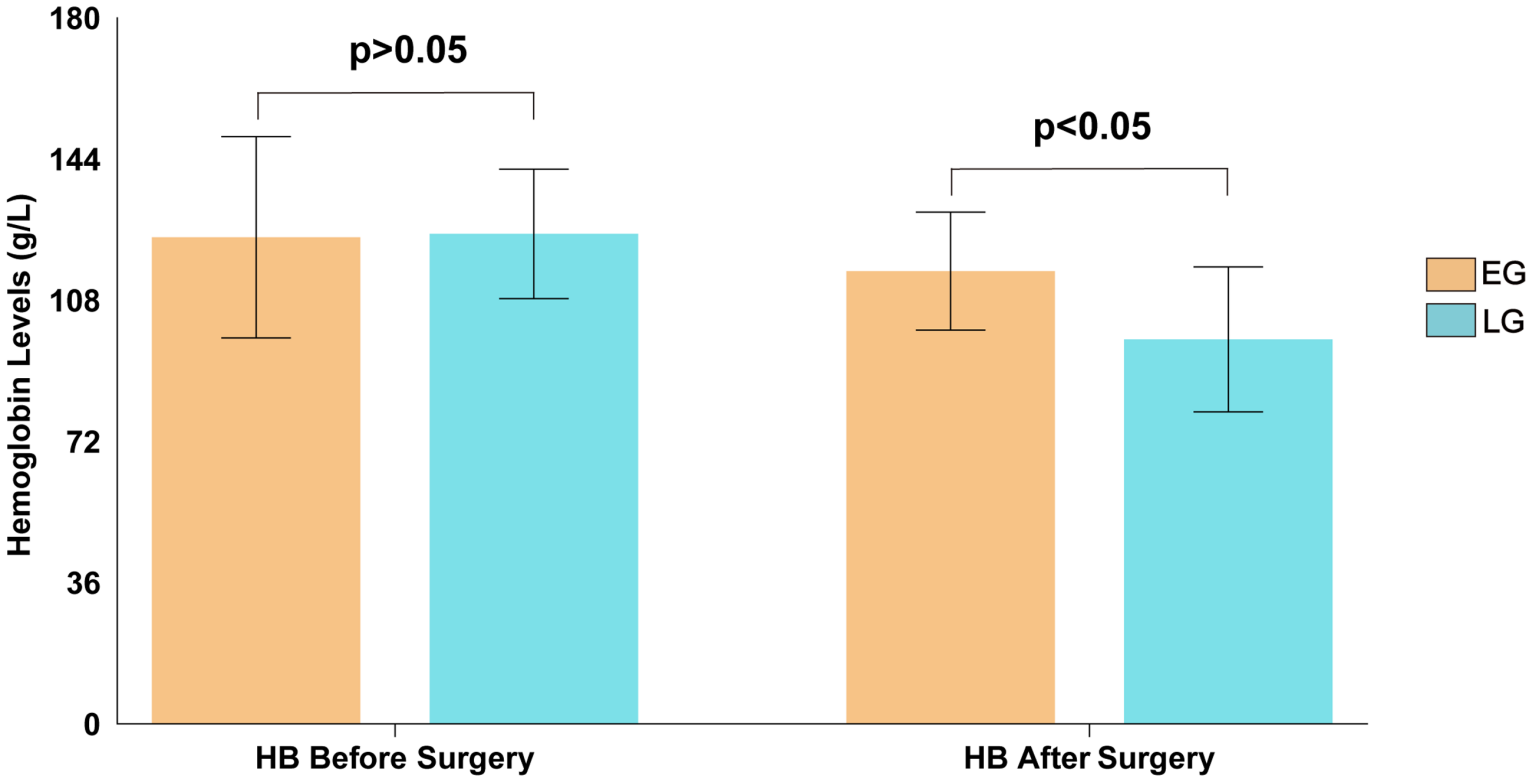
Comparison of hemoglobin levels between the two groups before and after surgery.

No significant difference was observed in preoperative hemoglobin levels between the two groups (126.10 ± 18.69 g/L vs. 124.93 ± 16.49 g/L, *t* = 0.431, *p* = 0.667). However, postoperative hemoglobin was significantly higher in the early group (115.43 ± 15.03 g/L vs. 98.04 ± 18.48 g/L, *t* = 6.609, *p* < 0.001) (Figure 4).

### 3.6 Complications

**Figure 5:**
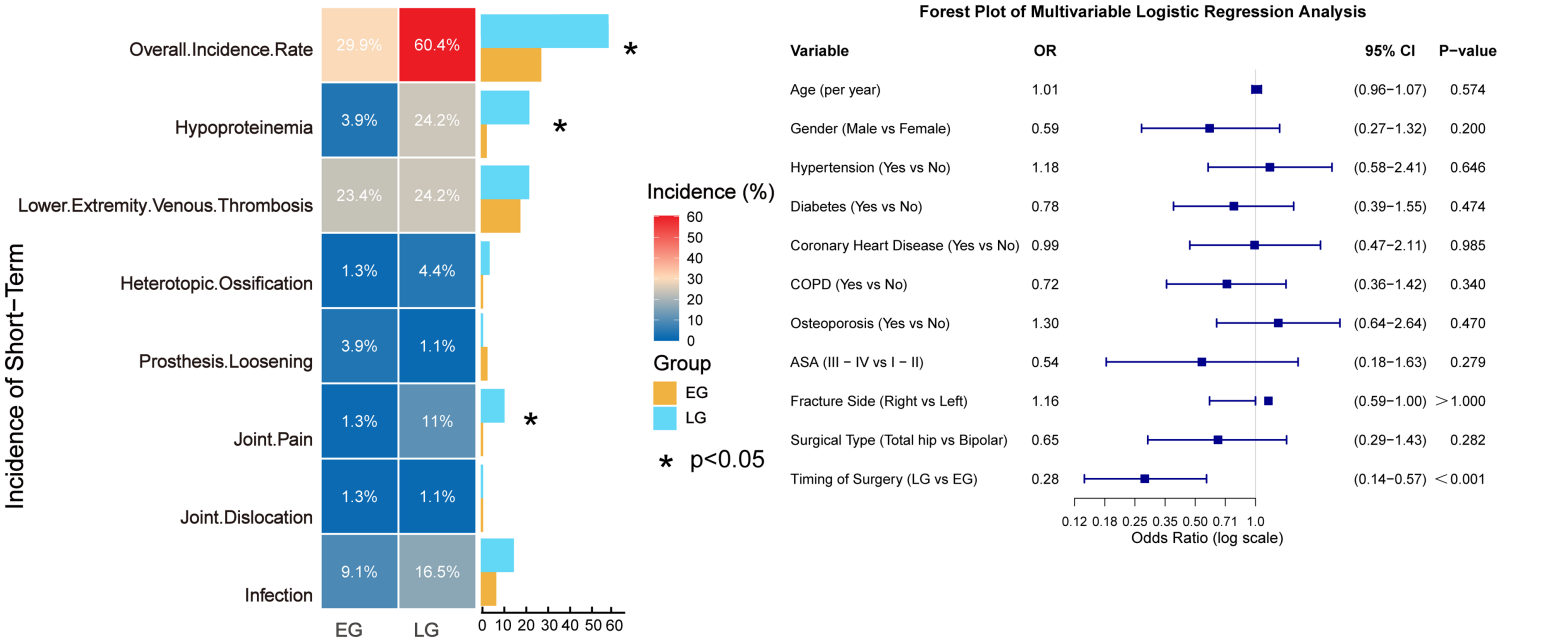
Incidence of short-term complications between the two groups. The model was adjusted for age, gender, ASA physical status classification, comorbidities, fracture side, surgical type.

The overall incidence of complications was significantly lower in the early group (29.9% vs. 60.4%, *χ^2^* = 15.670, *p* < 0.001) absolute risk reduction (95% CI) (30.5% (16.2 to 44.9%)) needed to treat (95%CI) (3 (2 to 6)). Specifically, the incidence of hypoalbuminemia (3.9% vs. 24.2%, *χ^2^* = 13.542, *p* < 0.001) absolute risk reduction (95% CI) (20.3% (10.5 to 30.1%)) needed to treat (95%CI) 5 (3 to 10) and joint pain (1.3% vs. 11.0%, *χ^2^* = 6.401, *p* = 0.012) absolute risk reduction (95% CI) (9.7% (2.8 to 16.6%)) needed to treat (95%CI) 10 (6 to 36) was significantly lower in the early group. No significant differences were found in the rates of deep vein thrombosis, heterotopic ossification, prosthesis loosening, dislocation, or infection (all *p* > 0.05). Multivariable logistic regression analysis revealed that, after adjusting for other covariates, the timing of surgery (OR 0.28, 95% CI 0.14–0.57, *p* < 0.05) was a significant independent risk factor for postoperative complications within 30 days. In contrast, age, sex, specific comorbidities, ASA classification, fracture side (left vs. right), and type of arthroplasty (bipolar hemiarthroplasty vs. total hip arthroplasty) did not demonstrate a statistically significant association with the risk of 30-day complications in this model (*p* > 0.05) (Table 4; Figure 5).

**Table 4:** Comparison of composite 30-day postoperative complications between two groups.

Complication	Early group (*n* = 77)	Delayed group (*n* = 91)	Absolute risk reduction (95% CI)	*p*-value	Needed to treat (95%CI)
Composite complications	23 (29.9%)	55 (60.4%)	30.5% (16.2 to 44.9%)	<0.001	3 (2 to 6)
Hypoalbuminemia	3 (3.9%)	22 (24.2%)	20.3% (10.5 to 30.1%)	<0.001	5 (3 to 10)
Hip joint pain	1(1.3%)	10 (11.0%)	9.7% (2.8 to 16.6%)	0.012	10 (6 to 36)
Infection	7 (9.1%)	15 (16.5%)	7.4% (−3.1 to 15.4%)	0.176	
Dislocation	1 (1.3%)	1 (1.1%)	−0.2% (−6.0 to 4.8%)	1.000	
Prosthesis loosening	3 (3.9%)	1 (1.1%)	−2.8% (−8.6 to 1.6%)	0.334	
Heterotopic ossification	1 (1.3%)	4 (4.4%)	3.1% (−3.2 to 9.6%)	0.376	
Deep vein thrombosis (DVT)	18 (23.4%)	22 (24.2%)	0.8% (−12.2 to13.4%)	1.000	

### 3.7 ICU admission, length of hospital stay

**Figure 6:**
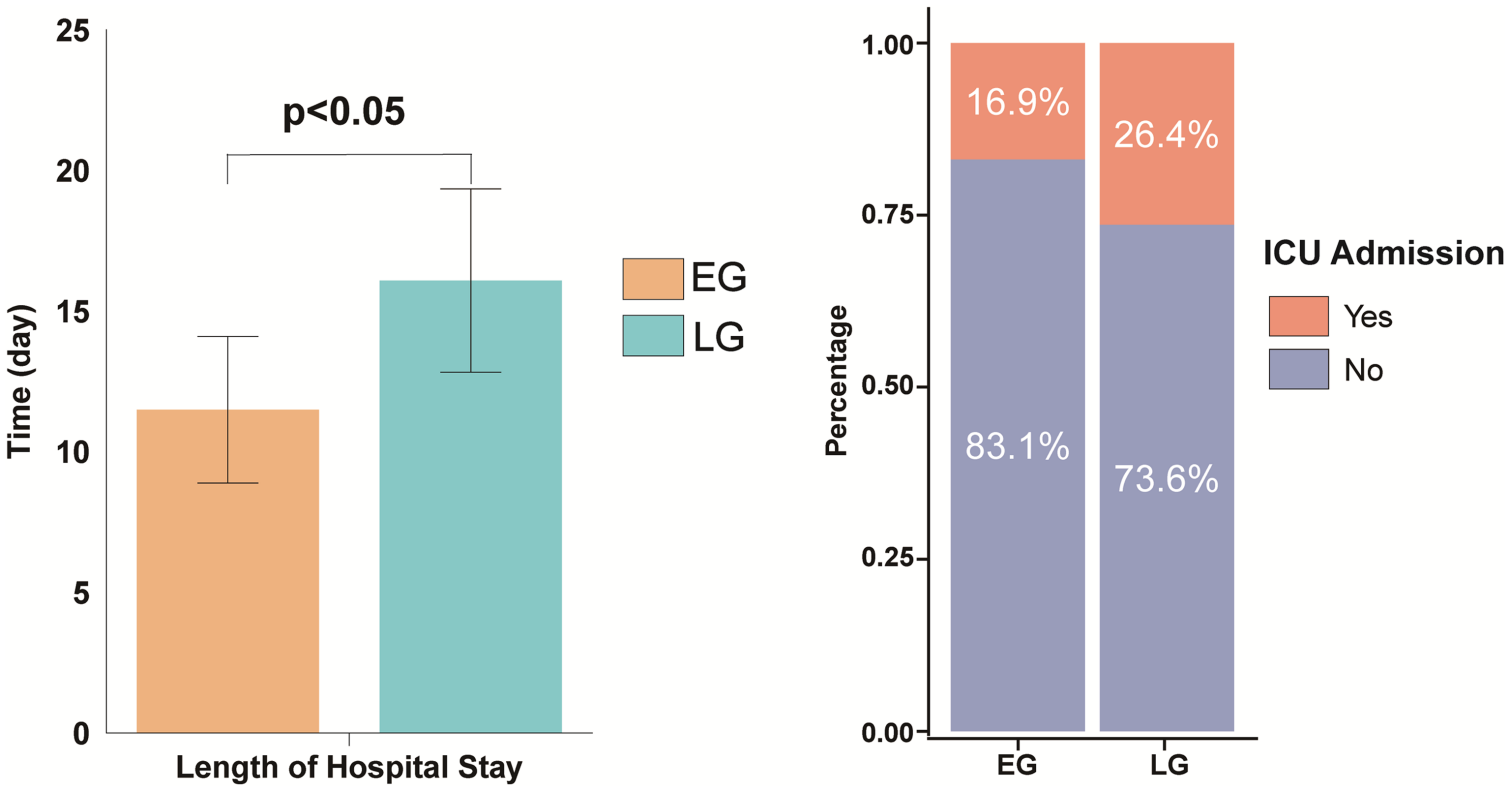
Comparison of postoperative length of hospital stay and ICU admission rates between the two groups.

There was no significant difference in ICU admission rates between the two groups (16.9% vs. 26.4%, *χ^2^* = 2.187, *p* = 0.139). However, the early group had a significantly shorter hospital stay (11.49 ± 2.60 days vs. 16.09 ± 3.26 days, *t* = −9.969, *p* < 0.001) (Figure 6).

### 3.8 Survival analysis

**Figure 7:**
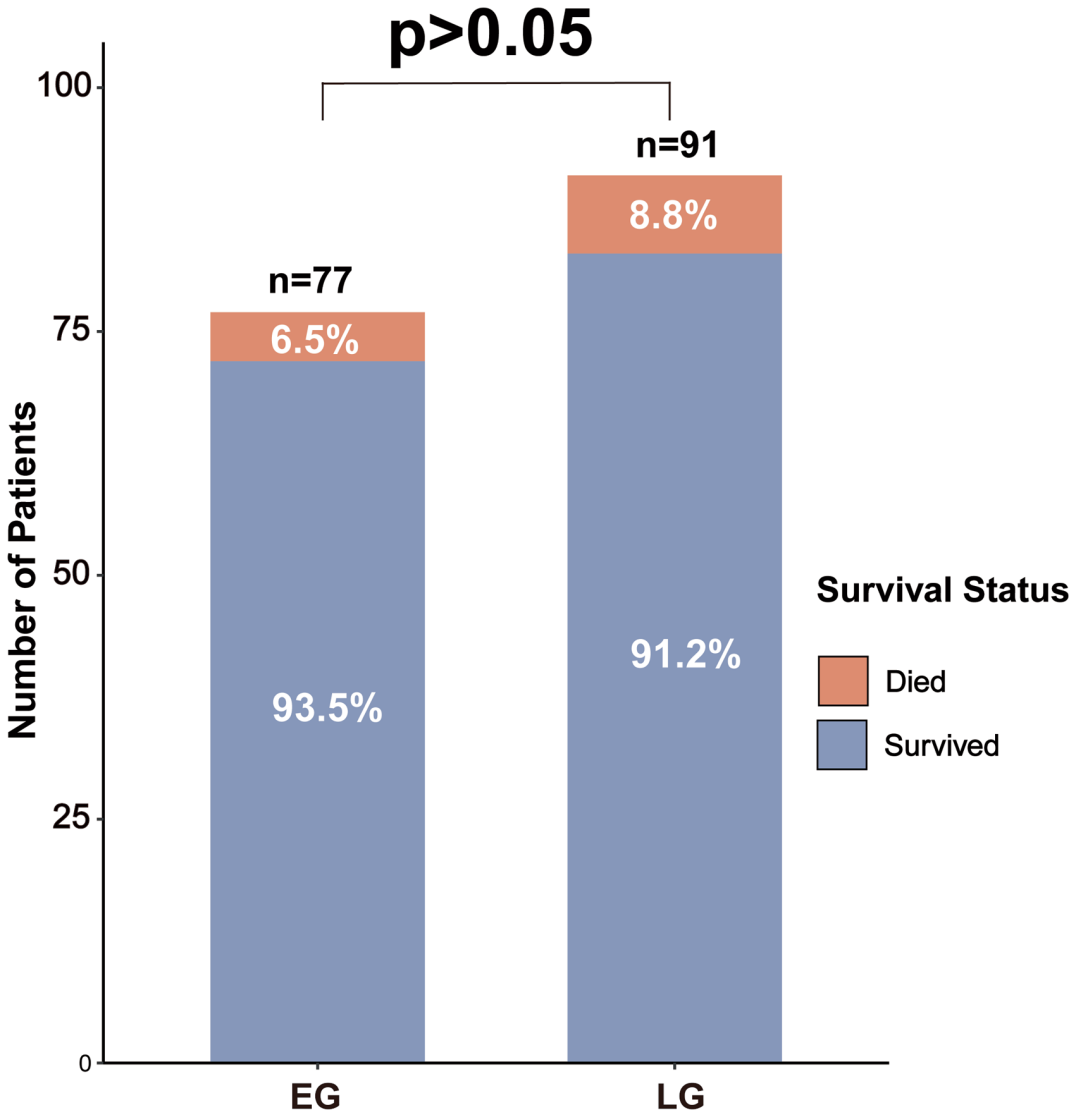
Comparison of 1-year survival rates between the two groups of patients after surgery.

There was no significant difference in mortality rates between the early group and the late group at the 1-year time point (6.5% vs. 8.8%, *χ^2^* = 0.308, *p* = 0.773) (Figure 7).

### 3.9 Hip function scores

**Figure 8:**
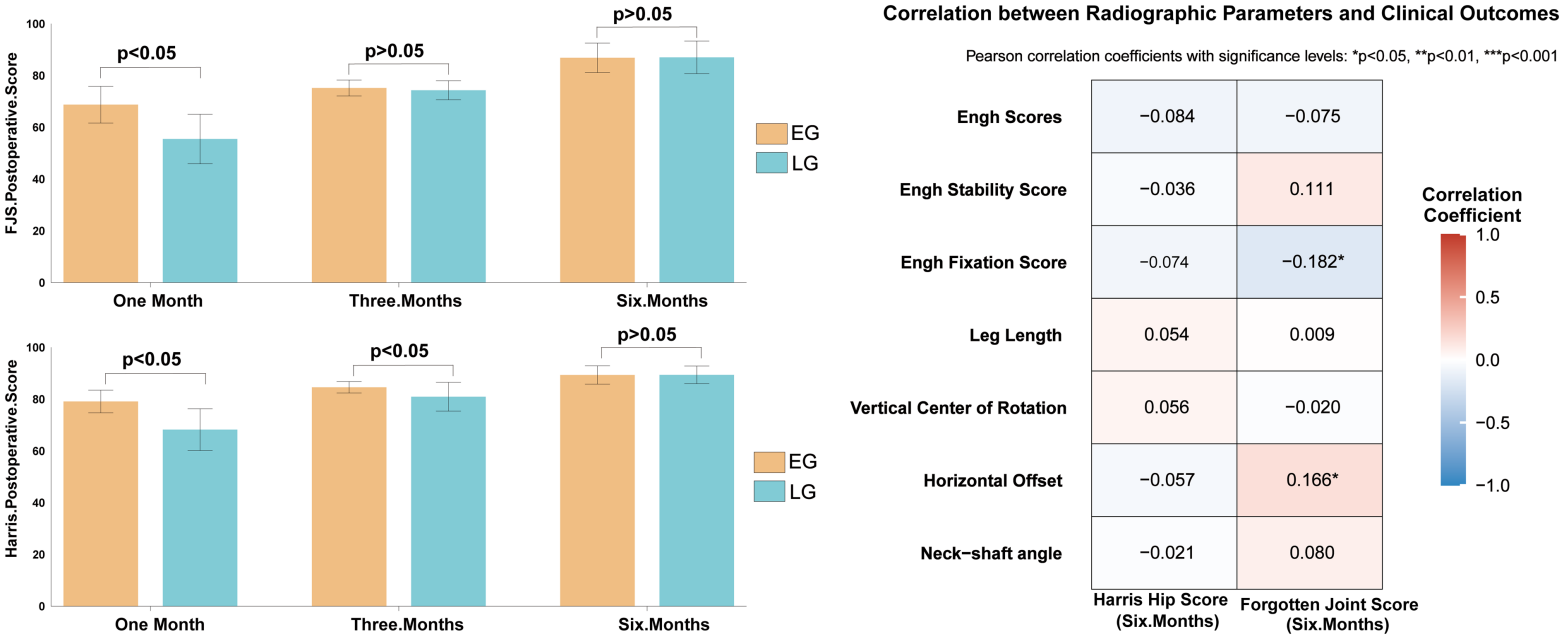
Comparison of Harris and FJS score between the two groups.

At 1 month postoperatively, the early group demonstrated significantly higher Harris Hip Scores (79.12 ± 4.37 vs. 68.24 ± 8.06, *t* = 11.090, *p* < 0.001) and Forgotten Joint Scores (FJS) (68.74 ± 7.10 vs. 55.46 ± 9.56, *t* = 10.308, *p* < 0.001). At 3 months, Harris scores remained higher in the early group (84.60 ± 2.20 vs. 80.95 ± 5.56, *t* = 5.753, *p* < 0.001), while FJS differences were no longer significant. At 6 months, both Harris and FJS scores were comparable between groups (all *p* > 0.05). Only the Engh fixation score (r = −0.182, *p* = 0.031) and postoperative horizontal offset (r = 0.166, *p* = 0.025) correlated significantly with the 6-month FJS. No radiographic parameters, including vertical center of rotation or leg length, showed a significant association with the 6-month HHS (Figure 8).

### 3.10 Pain assessment (VAS)

**Figure 9:**
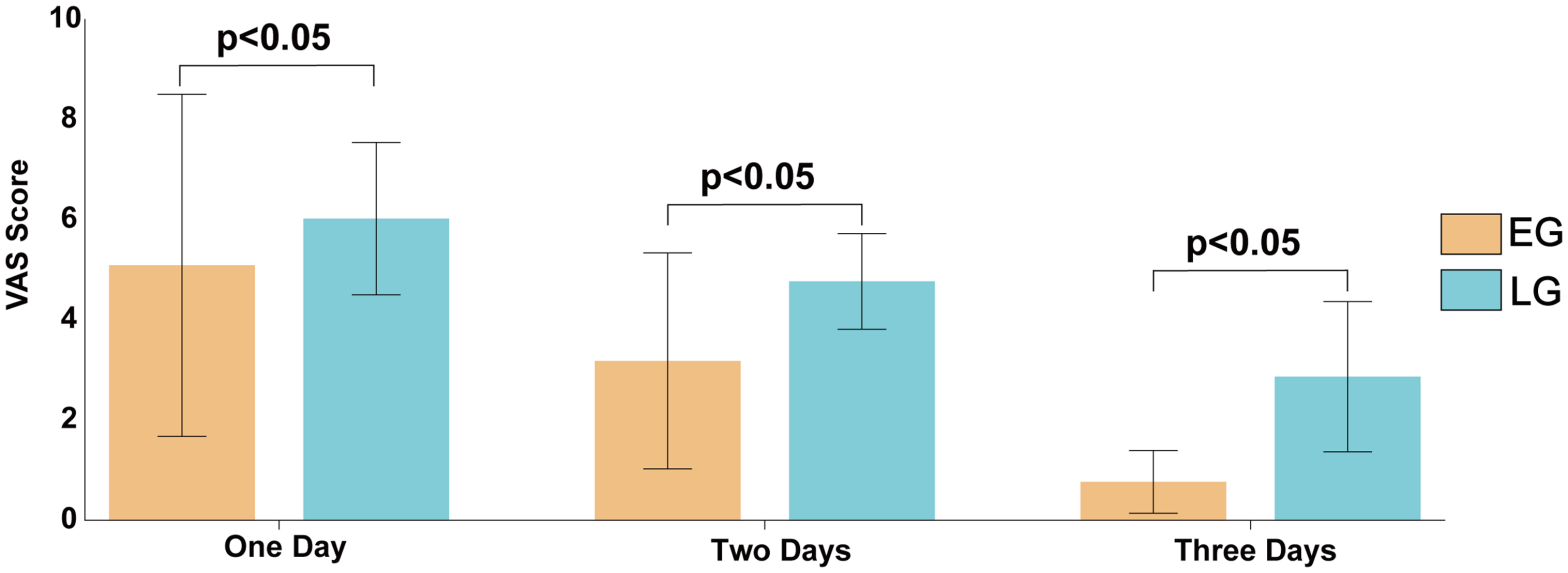
Comparison of VAS score between the two groups.

VAS scores were significantly lower in the early surgery group compared to the delayed group at all time points: Day 1: 5.09 ± 3.42 vs. 6.02 ± 1.52, *t* = −2.342, *p* = 0.029; Day 2: 3.18 ± 2.16 vs. 4.77 ± 0.96, *t* = −6.329, *p* < 0.001; Day 3: 0.77 ± 0.63 vs. 2.87 ± 1.50, *t* = −12.175, *p* < 0.001. These results indicate that patients in the early surgery group experienced significantly less pain during the acute post-injury period (Figure 9).

### 3.11 Psychological and social function

**Figure 10:**
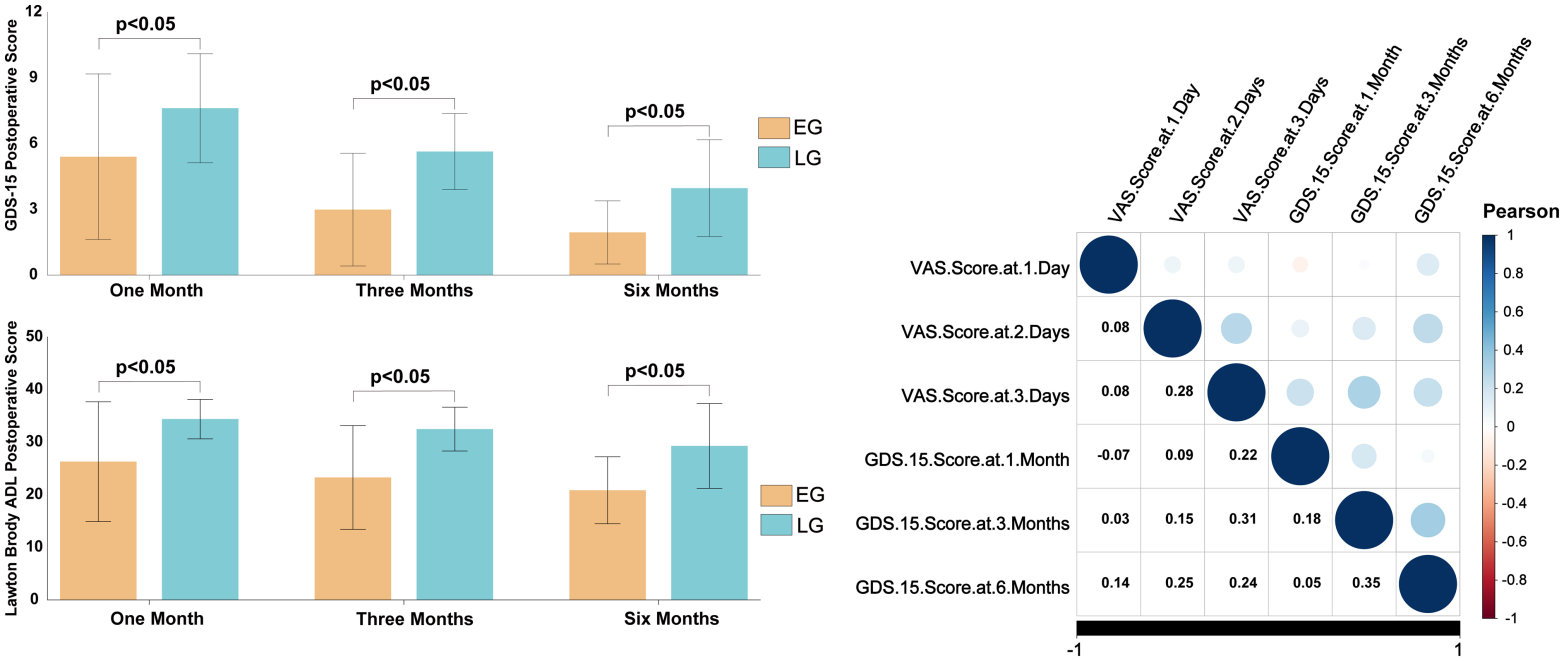
Comparison of GDS and Lawton score between the two groups.

GDS-15 Scores (lower scores indicate less depression):1 month: 5.40 ± 3.77 vs. 7.62 ± 2.49, *t* = −4.546, *p* < 0.001; 3 months: 2.99 ± 2.57 vs. 5.64 ± 1.74, *t* = −7.682, *p* < 0.001; 6 months: 1.95 ± 1.44 vs. 3.97 ± 2.21, *t* = −7.114, *p* < 0.001. Lawton-Brody IADL Scores (higher scores indicate greater independence):1 month: 26.29 ± 11.39 vs. 34.37 ± 3.75, *t* = −5.962, *p* < 0.001; 3 months: 23.27 ± 9.86 vs. 32.47 ± 4.17, *t* = −7.630, *p* < 0.001; 6 months: 20.84 ± 6.37 vs. 29.27 ± 8.06, *t* = −77.571, *p* < 0.001. Correlation analysis between VAS and GDS-15 scores indicated that the associations between pain and depressive symptoms were generally positive across most timepoints, though none reached statistical significance. Notably, the correlation between VAS on day 1 and GDS-15 at 1 month showed a weak negative trend, while all other examined pairings demonstrated weak positive correlations. These findings demonstrate that patients who underwent early surgery had significantly better psychological well-being and greater independence in daily activities throughout the follow-up period (Figure 10).

## 4 Discussion

Despite widespread consensus on the surgical management of femoral neck fractures in older adults, the optimal timing of hip arthroplasty remains controversial due to the absence of globally standardized guidelines. Current recommendations advocate for individualized decision-making within a limited preoperative window (2, 21). However, most prior studies have focused primarily on short-term morbidity, mortality, and functional outcomes, with limited emphasis on the integrated physical–psychological–social trajectory of recovery. Moreover, ethical constraints have largely precluded randomized controlled trials, leaving the evidence base reliant on observational data (3, 6–10).

In this retrospective cohort study, early surgical intervention (≤48 h) was associated with significantly lower overall complication rates, shorter hospital stays, and superior early functional recovery. These findings align with previous reports by Sharma et al. (22) and Anthony et al. (23), who demonstrated that surgical delay beyond 48–72 h was an independent risk factor for postoperative morbidity. Similarly, Sun et al. (24) observed that early surgery reduced the incidence of pulmonary infections, urinary tract infections, and deep vein thrombosis, while also decreasing hospital readmission rates. The beneficial effects of early surgery may be attributed to prompt clearance of fracture-site hematoma and necrotic tissue, thereby attenuating the systemic inflammatory response and oxidative stress cascade (25, 26).

Conversely, several studies have reported no significant difference in mortality or major complication rates between early and delayed surgery. Lim et al. (27) found no association between surgical timing and adverse events, while the multicenter HIP ATTACK trial (28, 29) concluded that accelerated surgery did not reduce the composite risk of mortality or major complications compared to standard care. These discrepancies may reflect heterogeneity in patient frailty, comorbidity burden, and perioperative management protocols. Indeed, early surgery without adequate multidisciplinary optimization may exacerbate physiological stress, particularly in frail or medically complex patients (30).

Our data suggest that early surgery confers short-term advantages without compromising mid-term functional outcomes. The early group exhibited higher Harris Hip Scores and Forgotten Joint Scores at 1 and 3 months postoperatively, with no significant differences observed at 6 months. This temporal pattern may be explained by preservation of periarticular soft-tissue integrity, prevention of capsular contracture, and early neuromuscular reactivation (31, 32). Moreover, early mobilization disrupts the “fracture–immobilization–complication” cycle, thereby reducing hospital-acquired deconditioning and cardiopulmonary morbidity (19, 33). Previous studies (34–36) have demonstrated that postoperative mortality in elderly patients is significantly associated with non-surgical factors such as delirium and malnutrition, indicating that early surgical intervention must be combined with multidisciplinary perioperative management to maximize survival benefits.

Postoperative psychological status is a critical determinant of functional recovery and quality of life in elderly patients with femoral neck fractures. Mood disorders such as anxiety and depression not only impair treatment compliance but may also establish a vicious cycle through psycho-physiological interactions, thus impeding the recovery process (37, 38). The data from this study demonstrate that VAS scores on post-injury days 1, 2, and 3 and GDS-15 scores at 1, 3, and 6 months postoperatively were significantly lower in the early-surgery group compared with the delayed-surgery group, suggesting that early hip arthroplasty can effectively shorten the duration of pain, alleviate negative emotions, and improve psychological well-being. Chronic pain is a well-established risk factor for depression in older adults (39), and our findings support the hypothesis that early pain relief attenuates depressive symptomatology, as evidenced by lower GDS-15 scores in the early group at all follow-up time points (40, 41).

Psychological well-being is increasingly recognized as a core domain of postoperative recovery. Prolonged preoperative waiting has been associated with heightened anxiety, fear of surgery, and reduced treatment adherence (11, 12). Our data demonstrate that early surgery significantly reduced depressive symptoms and improved instrumental activities of daily living (IADL) across the 6-month follow-up period. These findings are consistent with prior studies linking shorter hospital stays and early functional recovery with lower postoperative depression risk (42). The Lawton-Brody IADL scale, which assesses complex tasks such as medication management and community mobility, may serve as a sensitive proxy for social reintegration capacity (18).

Social participation is a neglected yet vital outcome in geriatric fracture care. Our results indicate that early surgery facilitates earlier resumption of community roles, likely via restoration of ambulatory independence and reduction in caregiver burden. However, the translation of functional recovery into social participation is modulated by multiple factors, including cognitive reserve, social support, and access to rehabilitation services (43–46). Therefore, maximizing social reintegration requires multidisciplinary coordination, psychological support, and continuity of care across the hospital–community–home spectrum (47).

Our findings further demonstrate that early surgical intervention significantly improves the medium- to long-term physical–psychological–social composite prognosis in older adults with femoral neck fractures. (i) Physical domain: Patients in the early surgery group consistently achieved higher hip function scores (Harris and FJS), likely due to prevention of disuse muscle atrophy and modulation of inflammatory-myogenic metabolic imbalance (48). (ii) Psychological domain: Early surgery was associated with lower long-term depression risk, as measured by GDS-15. The underlying mechanism may involve stabilization of the hypothalamic–pituitary–adrenal (HPA) stress axis, although cognitively impaired subgroups may require additional neurocognitive interventions to achieve similar benefits (49). (iii) Social domain: Lawton–Brody IADL scores improved significantly in the early group, indicating enhanced community participation, with the greatest social gains observed in the oldest-old cohort (50, 51). Collectively, these data support a tripartite vicious cycle in which physical disability and depressive symptoms synergistically drive social isolation, and early surgery effectively disrupts this loop by simultaneously addressing all three domains. Nevertheless, delayed surgery remains justifiable in selected high-risk patients, particularly when multidisciplinary optimization (e.g., cardiopulmonary tuning, anticoagulation bridging, or geriatric co-management) is required to mitigate perioperative risk. Thus, clinical decision-making should balance the benefits of “early rehabilitation” against the safety of “comprehensive preoperative preparation”, rather than rigidly adhering to a 48-h time window.

Limitations of this study include its single-center retrospective design, which may introduce selection bias and limit generalizability. Additionally, we did not stratify outcomes by implant type (hemiarthroplasty vs. total hip arthroplasty), which may confound the association between surgical timing and outcomes due to differences in operative complexity. Finally, long-term follow-up data (>1 year) were not available, precluding analysis of late complications such as periprosthetic fracture or aseptic loosening.

## 5 Conclusion

Early hip arthroplasty (≤48 h) should be considered the preferred strategy for medically stable elderly patients with femoral neck fractures, as it reduces complications, accelerates functional recovery, and improves psychological and social outcomes. However, individualized risk stratification remains essential, and delayed surgery may be justified in hemodynamically unstable or frail patients requiring multidisciplinary optimization. Future multicenter prospective trials should integrate biomarker profiling, frailty indices, and machine-learning models to develop precision-based surgical timing algorithms.

## Data Availability

The raw data supporting the conclusions of this article will be made available by the authors, without undue reservation.
